# A socio-ecological analysis of intimate partner violence among women of reproductive age in Nigeria: a multilevel analysis of data from 2008 to 2018 Nigeria demographic and health surveys

**DOI:** 10.1186/s12889-025-25220-8

**Published:** 2025-11-17

**Authors:** Ebenezer Kwesi Armah-Ansah, Seun Adegboyega Adejumo, Ekenedilichukwu Elvis Ezekobe, Ailin Jalili, Saad Ahmed, Ehinomen Oko-Oboh, Eugene Budu, Charity Oga-Omenka

**Affiliations:** 1 School of Public Health Sciences, Faculty of Health, University of Waterloo, Waterloo, Canada; 2 Institute of Human Virology, Abuja, Nigeria

**Keywords:** Emotional, Physical, Reproductive age, Sexual, SEM, Violence

## Abstract

**Introduction:**

Intimate partner violence (IPV) is a significant public health and human rights issue in Africa. Despite numerous initiatives and legislative measures, IPV continues to be deeply rooted in sociocultural norms, affecting both social and economic progress in Nigeria. Hence, this study utilizes the socio-ecological model to analyze trends and factors associated with IPV among women of reproductive age in Nigeria from 2008 to 2018.

**Methods:**

The study was an analytical cross-sectional study that utilized secondary datasets from the 2008, 2013, and 2018 Nigeria Demographic and Health Surveys. A weighted sample of 47,015 women of reproductive age was included in this study. The analysis was conducted using Stata. Multilevel regression analysis was applied, and the results were presented as adjusted odds ratios (aOR), along with 95% confidence intervals (CIs) and p-values to indicate the statistical significance of the findings.

**Results:**

The overall prevalence of IPV in Nigeria from 2008 to 2018 was 25.94% [95% CI: 25.11%-26.80%]. The average prevalence of emotional violence was 21.82% [95% CI: 21.03%-22.63%], whereas that of physical violence was 11.35% [95% CI: 10.86–11.85%], and sexual violence was 3.53% [95% CI: 3.29%-3.80%]. Significant risk factors associated with IPV included primary education [aOR = 1.17; 95% CI = 1.08–1.26], cohabiting [1.40; 95% CI = 1.23–1.59]; working [aOR = 1.30; 95% CI = 1.23–1.59], having four or more births [aOR = 2.08; 95% CI = 1.87–2.32], exposed to mass media [aOR = 1.17; 95% CI = 1.10–1.23], belonged to Hausa ethnic group [aOR = 1.65; 95% CI = 1.43–1.89], women whose partners had primary education [aOR = 1.21; 95% CI = 1.12–1.31], those in a polygamous family type [aOR = 1.31; 95% CI = 1.24–1.39], those who lived in North East [aOR = 1.37; 95% CI = 1.25–1.50], those who lived communities with high literacy level [aOR = 1.17; 95% CI = 1.04–1.32] and those who were in 2018 survey year [aOR = 1.47; 95% CI = 1.37–1.57].

**Conclusion:**

The research indicates that intimate partner violence continues to be a significant public health concern in Nigeria, with approximately 25.94% of women of reproductive age experiencing IPV from 2008 to 2018, reaching a peak of 33.24% in 2018, which signals a troubling upward trend. The most common type of violence reported was emotional abuse, impacting nearly 22% of women. Major risk factors identified include lower educational attainment for both women and their partners, cohabitation, employment status, higher numbers of children, access to mass media, identification as Hausa, involvement in polygamous family arrangements, residing in the Northeast region, and living in areas with higher literacy rates. These findings highlight the multi-faceted factors contributing to IPV. To effectively address this issue, it is necessary to implement targeted, multi-faceted strategies that focus on educational inequities, economic conditions, cultural practices, and regional disparities, with a strong focus on prevention to halt the rising trend of violence.

## Background

Violence against women of reproductive age represents a widespread manifestation of gender inequality and constitutes a significant infringement of human rights, with serious implications for public health [1–4]. Sustainable Development Goal (SDG) 5 aims to eliminate all forms of violence against women by 2030, with Target 5.2 focusing specifically on IPV to eradicate violence in both public and private spheres, including trafficking and exploitation [5, 6]. Intimate partner violence (IPV) is the most prevalent form, disproportionately impacting women in low-income nations [4, 7]. On a global scale, approximately one in three women will face IPV at some point in their reproductive age [8]. Intimate partner violence refers to any actions within a close relationship that inflict physical, emotional, or sexual harm [9], with societal acceptance of such violence varying significantly by region [10].

Intimate partner violence does not only undermine women’s confidence; it diminishes their productivity and ability to contribute to societal development, and it extensively affects their control over sexual and reproductive health, sexual, physical, mental, and emotional well-being, and the well-being of their families and communities and the economic and social structures of societies [11–13]. The repercussions on reproductive health are particularly grave, as victims of IPV experience increased risks of unplanned pregnancies, complications during pregnancy, miscarriage, low birth weight, premature births, and sexually transmitted infections [14–18]. Long-term health issues often endure well after the violence has ceased, leading to chronic pain and gastrointestinal problems [4], impairing women’s ability to care for their children [19], and disrupting breastfeeding practices [15, 20, 21].

Additionally, physical, emotional, and psychological violence may lead to injuries and disabilities, as well as significant emotional distress. Victims may experience attempted or completed suicide, develop post-traumatic stress disorder, and suffer from depression, anxiety, and low self-esteem. Furthermore, they may engage in negative behaviours, such as substance abuse, which can necessitate medical intervention [9, 11, 22, 23].

The WHO African region, primarily encompassing sub-Saharan Africa (SSA) countries, reports an IPV prevalence of 36.6%, one of the highest globally, only surpassed by South-East Asia (38%) and the Eastern Mediterranean (37%) [5, 24]. These three regions account for 37% of women who have ever had a partner and have experienced IPV, significantly higher than the global average of 30% [25]. Within the African region, higher prevalence of IPV against women of reproductive age is observed in East Africa (44.1%) and Central Africa (49.3%) [24]. Physical and sexual violence rates are alarmingly high, impacting up to 30.58% and 12.6% of women, respectively [26]. At the country level, lower prevalence of IPV against women of reproductive age is reported in Comoros (10.8%) and the highest in Sierra Leone (59.9%) [26].

In Nigeria, IPV rates differ across geopolitical zones; the South-East region exhibits the highest rate of IPV at 78.8%, 41% in the South-South, and 29% in the South-West [27, 28]. These variations are influenced by factors such as levels of education, economic dependency, infertility issues, and cultural gender norms [29–31]. In the southern regions, while higher educational attainment among women may enhance conflict resolution skills, it can also be perceived as a challenge to male authority, which may, in turn, lead to increased rates of intimate partner violence (IPV) [32]. Other contributing elements include socioeconomic status (income, employment), marital structures (e.g., polygyny), decision-making power, religious beliefs, partner literacy, and community perceptions of IPV [33–35]. The literature indicates that the burden of intimate partner violence (IPV) has worsened due to the COVID-19 pandemic [36, 37].

The severe health effects and societal consequences of IPV have prompted policymakers, program implementers, and civil society to create comprehensive responses [38–40]. This dedication is evident in the increased programming and larger budget al.locations aimed at preventing and addressing IPV [30]. Legislative measures have also been taken, with various states in Nigeria passing progressive laws, including Ekiti State’s Gender-Based Violence Prohibition Law of 2011 and Lagos State’s Domestic Violence Prohibition Law of 2007 [41, 42]. Laws alone are not enough; systemic and contextual obstacles limit their efficacy [43, 44].

Even with these initiatives, IPV continues to be a problem deeply rooted in sociocultural norms, affecting both social and economic progress in Nigeria [28, 30, 32, 45]. To effectively tackle these complex drivers, it is essential to go beyond one-off cross-sectional studies. Furthermore, analyzing a decade-long trend of IPV and its associated predictors is crucial for creating targeted interventions aimed at addressing its root causes and alleviating its devastating effects on individuals, families, and communities.

This study utilizes the socio-ecological model (SEM) to analyze IPV trends in Nigeria over the last decade, pinpointing significant predictors at individual, interpersonal, and community levels. Employing SEM will enable us to better understand the various risk factors that contribute to IPV in Nigeria, which in turn aids in creating more focused, evidence-driven prevention and intervention plans, improving their effectiveness across different structural and community contexts. Also, the findings from this will promote gender equity in Nigeria and add to the existing body of literature on IPV.

### Theoretical framework

This current study used the socio-ecological model (SEM) to investigate the trends and factors associated with IPV among women of reproductive age in Nigeria from 2008 to 2018. The phenomenon of IPV is a complex and diverse issue shaped by a range of interconnected individual, interpersonal, and community elements, necessitating a holistic approach grounded in the socio-ecological model (SEM) [46]. This model assists educators and researchers in understanding the intricate realities surrounding IPV [47]. Bronfenbrenner [48] introduced the SEM to emphasize the significance of the social environment on individual behaviours by analyzing the interactions among individual, relational, community, and societal elements. The SEM is illustrated through a series of concentric circles that represent various levels within the environment [49]. The WHO has endorsed the use of an SEM to comprehend nearly all types of interpersonal violence interventions and prevention, framing violence as a public health issue [50].

The WHO’s model categorizes four social levels, including individual, relationship, community, and societal, which correspond with Bronfenbrenner’s hierarchical structure [49]. The SEM has previously been applied in Kenya in 2018 [46, 51], Somalia [52], and a meta-analysis in sub-Saharan Africa [49]. These studies collectively underscore the necessity of considering various levels of SEM when understanding and preventing IPV, leading to the development of targeted interventions and prevention strategies. Heise [53] was a pioneer in applying the SEM to analyze violence against women, reviewing both local and cross-cultural literature to investigate the factors associated with IPV perpetration and victimization. Also, Hong et al. [54] examined wife abuse in South Korea through the ecological model, emphasizing how patriarchal views, a culture of drinking, and weak legal enforcement contribute to ongoing violence. In a similar vein, Willie and Kershaw [55] employed an ecological analysis to explore the connection between gender inequality and IPV in the United States, highlighting how societal norms and economic disparities affect the prevalence of IPV. These investigations illustrate how the ecological model effectively captures the complex and context-dependent aspects of IPV.

Further uses of the ecological model showcase its adaptability in analyzing IPV across various contexts [56]. Through these varied applications, the SEM provides a strong foundation for creating targeted prevention and intervention efforts that tackle IPV across multiple societal levels. We utilized the SEM due to its public health relevance and its ability to organize multifactorial risk factors into interconnected levels across individual-level, interpersonal-level, and community-level factors. While most research focuses on risk factors at the individual level, it is crucial to recognize that these risk factors also exist at all levels of the SEM [46].

Ultimately, viewing IPV through this framework enhances our understanding of its complexities and provides a foundation for effective prevention and intervention strategies, which require collaborative efforts across different sectors to challenge deep-rooted norms and foster healthier relationships within communities [57, 58].

## Materials and methods

Data from the 2008, 2013, and 2018 Nigeria Demographic and Health Surveys (NDHS) were used for this study. The NDHS, a national survey, is undertaken on a five-year basis to offer an important repository of data for analysis and examination of crucial aspects of fertility levels, maternal and child health, family planning, maternal and child nutrition, and newborn and child mortality in the country [59]. The survey employs a two-stage stratified sampling technique, and the data captured is nationally representative. In the first stage, clusters were selected from urban and rural locations across the country. In the second stage, households were selected from predefined clusters based on the Population and Housing Census of the Federal Republic of Nigeria (NPHC). The final reports contain information on the methodologies used in each of the survey rounds [60]. The dataset is freely available for download at https://dhsprogram.com/data/dataset/Nigeria_Standard-DHS_2018.cfm?flag=1. For this study, only women who were either married or cohabiting (*n* = 17,853 in 2008, 20,849 in 2013, and 8,313 in 2018) were included. After the addition of all data sets, the total number of respondents was 47,015. The study relied on Strengthening the Reporting of Observational Studies in Epidemiology (STROBE) statements [61].

### Inclusion criteria

For this study, we used a women’s file, which included a total weighted sample of 47,015 eligible women aged from 15 to 49 years from the selected clusters. This included all women of reproductive age who were currently married and cohabiting. The analysis was restricted to those women who had complete information on PV and other variables used in the study. Women who reported experiencing physical, sexual, or emotional violence in the past year were classified as having experienced IPV.

### Variables

#### Dependent variable

In the NDHS, violence information was obtained from ever-married and cohabiting women on violence committed by their current and former spouses/partners [62]. Based on literature [63–66], the dependent variable was generated from women’s responses on if she had experienced intimate partner violence in the 12 months prior to the survey. This was classified as physical violence, sexual violence, and emotional violence. The NDHS included a range of questions pertinent to physical violence. This included ever (1) being pushed/shook/thrown something; (2) being slapped; (3) being punched/hit by something; (4) being kicked; (5) having an arm twisted; (6) having a bruise because of the husband’s actions; and (7) having injuries, sprains, dislocations, or burns.

Emotional IPV was assessed from responses to the following questions: Have you ever (1) been humiliated by husband/partner, (2) been threatened with harm by husband/partner, (3) been insulted or made to feel bad by husband/partner, or (4) experienced any other emotional violence.

On sexual violence, the following questions were used: whether the partner ever (1) physically forced the respondent into unwanted sex, (2) forced her into other unwanted sexual acts, and (3) the respondent has been physically forced to perform sexual acts she didn’t want to.

To analyze physical, emotional, and sexual violence, a binary variable was established to indicate whether a participant had encountered any of these types of violence in the past year, coding responses of ‘never’ as ‘No,’ while responses of ‘often’ and ‘sometimes’ were combined and coded as ‘Yes.’ The “Yes” responses were coded ‘1,’ while “No” responses were coded ‘0.” The focus of the analysis was restricted to IPV experiences within the last year, and to minimize any biases that could arise from considering lifetime experiences, the response category ‘yes, but not in the last 12 months’ was dropped from the analysis [67].

### Independent variables

This study included seventeen independent variables, based on previous research [5–29] and their availability in the DHS. These variables associated with IPV are present at different levels of SEM. Using the SEM, these seventeen independent variables were grouped as individual-level factors, interpersonal-level factors, and community-level factors [68].

### Individual-level factors

Individual-level factors included age (15–19, 20–24, 25–29, 30–34, 35–39, 40–44, 45–49); education (no formal education, primary level, secondary/higher); marital status (married, cohabitation); employment status (not working, working); health insurance coverage (no, yes); parity (zero, one, two, three, or four or more births); religion (Christian, Islam, traditional, no/other); ethnicity (Fulani, Hausa, Igbo, Yoruba, others); mass media exposure (yes, no); and wealth index (poorest, poorer, middle, richer, richest).

Mass media was a composite variable that included listening to the radio, watching television, and reading newspapers and magazines. These variables had the same response categories: “not at all,” “less than once a week,” and “at least once a week.” Based on literature, the answer options were divided into “no”, which indicated no mass media exposure (not at all), and “yes,” which signified mass media exposure (less than once a week and at least once a week) [69–71].

### Interpersonal-level factors

Interpersonal-level factors included partner’s education (no formal education, primary level, or secondary/higher); partner’s employment status (not working or working); family type (monogamous or polygamous); and living with a partner (no or yes).

### Community-level variables

The community-level variables included four categories: place of residence (urban, rural); geopolitical zone (North Central, North East, North West, South West, South East, and South South); community literacy level (low, medium, high); community socioeconomic status (low, medium, high); and survey year (2008, 2013, 2018). The geopolitical zone of North Central included Benue, Kogi, Kwara, Nasarawa, Niger, Plateau, and Abuja states, whereas North East included Adamawa, Bauchi, Borno, Taraba, and Yobe states. Jigawa, Kaduna, Kano, Katsina, Kebbi, Sokoto, and Zamfara states were categorized as North West, while Oyo, Ogun, Ekiti, Lagos, Ondo, and Osun states were grouped as South West. The Southeast geopolitical zone included Abia, Anambra, Ebonyi, Enugu, and Imo states, and the South-South geopolitical zone had Akwa Ibom, Bayelsa, Cross River, Delta, Edo, and Rivers states. The community literacy level was calculated based on the proportion of women who could read and write or could not read and write at all. The community’s socioeconomic level was determined by the employment, income, and education of the women who lived there.

We used principal component analysis to determine the number of women who were jobless, uneducated, and impoverished. A standardized score was created, with a mean of 0 and a standard deviation. The scores were then divided into three terciles: 1 (least disadvantaged), 2 (middle disadvantaged), and 3 (most disadvantaged), with tercile 1 reflecting a better socioeconomic situation and tercile 3 signifying a poorer one [70, 72].

### Statistical analyses

**Fig. 1:**
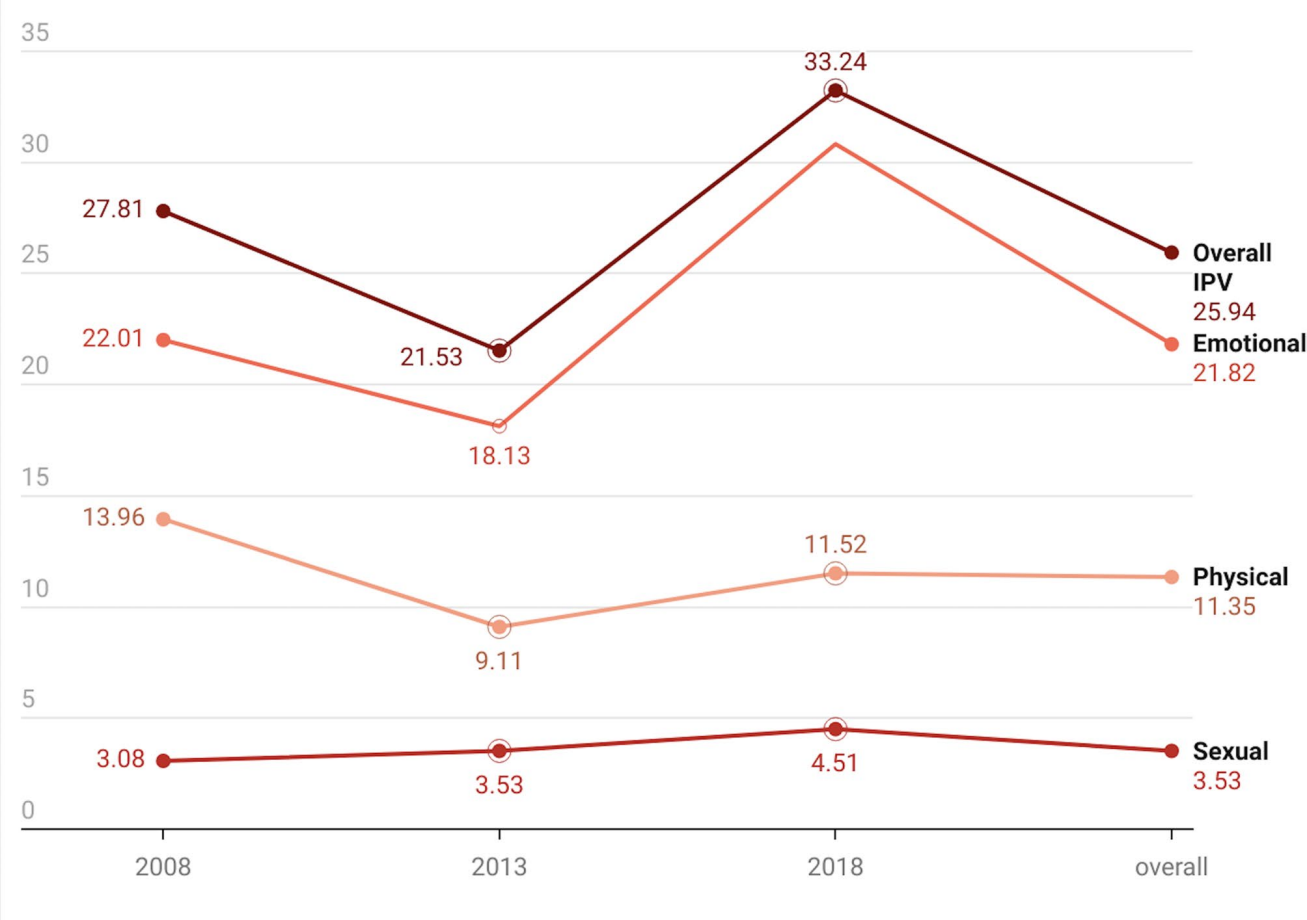
Trends in prevalence of IPV from 2008–2018

The study used Stata version 14.2 to analyze the data from three rounds of NDHS. Three levels of analyses (univariate, bivariate, and multivariate) were conducted. At the univariate level, graphical representations were used to illustrate the trends and prevalence of IPV in Nigeria. Figure 1 presents the trends in types of IPV (physical, sexual, and emotional) across the three survey years using a line chart. It also shows the overall IPV trend during the same period. Next, a bivariate level analysis of the percentage of IPV across the independent variables was calculated (Table 1). Specifically, the explanatory variables’ weighted frequencies and percentages were reported with p-values, which were derived from a chi-square of fitness. Figure 3 shows the same patterns visually, comparing IPV rates across different demographic groups. Statistically significant variables in the bivariate analysis were moved to the multilevel regression model (Table 2). Adjusted odds ratios (aOR) with 95% confidence intervals (CI) were used to present the results of the multilevel regression. Multicollinearity was checked among the independent variables using the variance inflation factor (VIF); no evidence of high collinearity was detected (mean VIF = 1.81, maximum = 3.84, minimum = 1.04). The highest VIF was observed for community literacy level, and the lowest for health insurance coverage.

In the multilevel regression, five models (Models 0-IV) were constructed (Table 2). The first model (Model 0) was the empty model, which had no independent variable. Models I, II, and III were based on the individual-level, interpersonal-level, and community-level variables, respectively. Model IV was the final and complete model that contained all the independent variables. Akaike’s Information Criterion (AIC) tests were also used to compare the models. The model is better and more like the unidentified real data generation process when the AIC is smaller. As a result, the model with the lowest AIC in the study was used for the discussion. Hence, Model IV was selected for predicting the factors associated with IPV in Nigeria. Figure 3 shows a forest plot of adjusted odds ratios and 95% confidence intervals from Model IV, highlighting key factors linked to IPV. Significance levels: *p* < 0.05, *p* < 0.01, *p* < 0.001. The data were weighted using v005/1,000,000 and analyzed using Stata’s survey set command (svy) to account for the survey’s complexity and the results’ generalizability.

## Results

### Prevalence of IPV from 2008 to 2018

The overall IPV prevalence decreased from 27.81% in 2008 to 21.53% in 2013. However, there was a resurgence in IPV prevalence to 33.24% in 2018. Overall, the weighted average IPV prevalence in Nigeria from 2008 to 2018 was 25.94% [95% CI: 25.11%−26.80%].

The 2018 NDHS was observed to have the highest prevalence of sexual IPV at 4.51% [95% CI: 4.01%−5.07%] and emotional IPV at 30.83% [95% CI: 29.48%−32.21%], while the 2008 NDHS had the highest prevalence of physical violence at 13.96% [95% CI: 13.29%−14.66%]. The average prevalence of physical violence was 11.35% [95% CI: 10.86–11.85%], whereas that of sexual violence was 3.53% [95% CI: 3.29%−3.80%] and emotional violence was 21.82% [95% CI: 21.03%−22.63%] (Fig. 1).

### Source: NDHS 2008, 2013, and 2018

#### Socio-demographic characteristics of women of reproductive age 2008–2018

The study’s weighted sample included 47,015 women of reproductive age, with the largest group (22.8%) being aged 25–29 years. While the majority of the participants had no formal education (43.6%), their partners (45.1%) had secondary/higher education. Most of the respondents were married (97.1%), employed (71.4%), their partners were employed (98.6%), were not covered by health insurance (97.9%), belonged to a monogamous family type (77.5%), were living with a partner (90.4%), were exposed to mass media (62.7%), were affiliated with the Islam religion (55.0%), and belonged to other ethnic groups (63.6%). Almost half of the respondents (47.3%) had four or more births, while 21.4%, 62.7%, and 31.0% were in the poorest wealth quintile, rural areas, and the Northwest, respectively. The majority of the respondents (31.0%, 40.5%, and 52.1%) were in the Northwest geopolitical zone, with low community literacy levels and low socioeconomic status, respectively, whereas 45.0% were in the 2013 survey year (Table 1).

**Table 1:** Socio-demographic characteristics of women of reproductive age 2008–2018 (*N* = 47,015)

Variables	Weighted *N*	Weighted (%)	χ2 (*p*-value)
Individual-level variables			
Age			
15–19	3,761	8.0	**178.33**, *p* < 0.001
20–24	7,722	16.4	
25–29	10,714	22.8	
30–34	8,701	18.5	
35–39	7,050	15.0	
40–44	4,807	10.2	
45–49	4,260	9.1	
Education			
No education	20,492	43.6	**390.84**, *p* < 0.001
Primary	9,249	19.7	
Secondary/Higher	17,274	36.7	
Marital Status			
Married	45,634	97.1	59.21 *p* < 0.001
Cohabitation	1,381	2.9	
Employment status			
Not working	13,434	28.6	**257.61**, *p* < 0.001
working	33,581	71.4	
Health insurance coverage			
No	46,033	97.9	**20.10**, *p* < 0.001
Yes	982	2.1	
Parity			
Zero	4,070	8.7	**319.53**, *p* < 0.001
One birth	6,658	14.1	
Two and three births	14,067	29.9	
Four or more births	22,220	47.3	
Mass Media Exposure			
No	17,553	37.3	**24.34**, *p* < 0.001
Yes	29,462	62.7	
Religion			
Christian			**5.72**, *p* < 0.001
Islam			
Traditional			
No/other			
Wealth index			
Poorest	10,060	21.4	**199.98**, *p* < 0.001
Poorer	9,545	20.3	
Middle	8,580	18.3	
Richer	8,979	19.0	
Richest	9,851	21.0	
Ethnicity			
Fulani	1,895	4.0	**284.68**, *p* < 0.001
Hausa	7,094	15.1	
Igbo	3,506	7.4	
Yoruba	4,636	9.9	
Others	29,884	63.6	
Interpersonal-level variables			
Partner’s education			
No education	17,059	36.3	**398.08** *p* < 0.001
Primary	8,764	18.6	
Partner Employment status			
Not working	663	1.4	**2.22** *p* = 0.14
working	46,352	98.6	
Family type			
Monogamous	36,457	77.5	**24.92**, *p* < 0.001
Polygamous	10,558	22.5	
Living with partner			
No	4,528	9.6	**25.99**, *p* < 0.001
Yes	42,487	90.4	
***Community-level variables***			
Place of residence			
Urban	17,551	37.3	**17.85**, *p* < 0.001
Rural	29,464	62.7	
Geopolitical Zone			
North Central	6,560	14.0	**7.35**, *p* < 0.001
North East	7,072	15.0	
North West	14,576	31.0	
South West	8,844	18.8	
South East	4,493	9.6	
South South	5,470	11.6	
Community literacy level			
Low	19,039	40.5	**313.03**, *p* < 0.001
Medium	13,979	29.7	
High	13,998	29.8	
Community socioeconomic status			
Low	24,510	52.1	**30.15**, *p* < 0.001
Medium	7,093	15.1	
High	15,411	32.8	
Survey year			
2008	17,544	37.3	**447.26** *p* < 0.001
2013	21,158	45.0	
2018	8,312	17.7	

A *p*-value below 0.05 shows statistical significance

### Source: NDHS 2008, 2013, and 2018

The data showed that age (*p* < 0.001), educational level (*p* < 0.001), partner’s educational level (*p* < 0.001), marital status (*p* < 0.001), employment (*p* < 0.001), ethnicity (*p* < 0.001), parity (*p* < 0.001), mass media (*p* < 0.001), family type (*p* < 0.001), health insurance coverage (*p* < 0.001), living with partner (*p* < 0.001), religion (*p* < 0.001), wealth (*p* < 0.001), place of residence (*p* < 0.001), geopolitical zone (*p* < 0.001), community literacy level (*p* < 0.001), community socioeconomic status (*p* < 0.001), and survey year (*p* < 0.001) were statistically associated with IPV (Table 1).

### Prevalence of IPV among women of reproductive age in Nigeria (2008–2018)

**Fig. 2:**
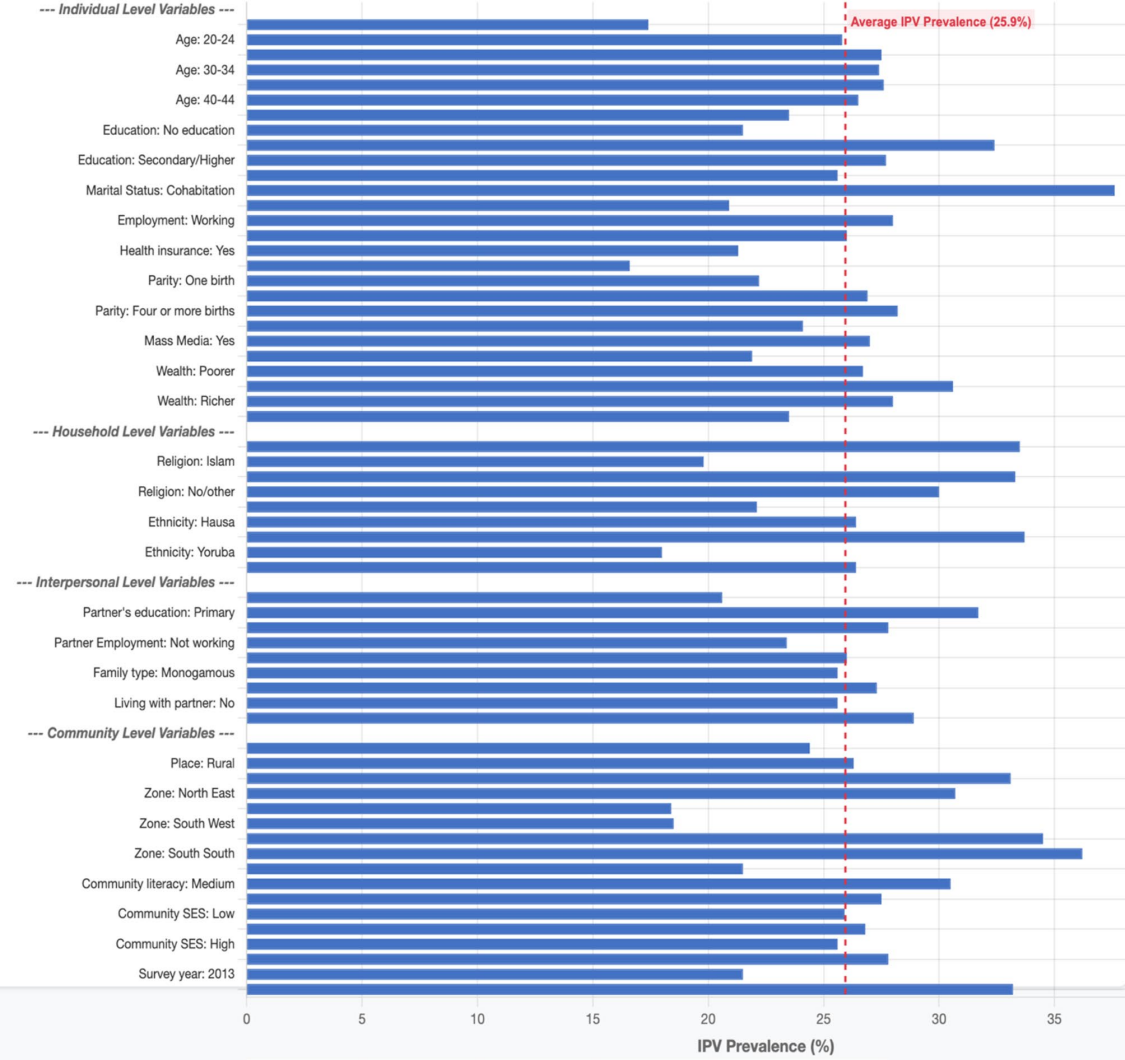
Prevalence of IPV among women of reproductive age in Nigeria (2008–2018)

In Fig. 2, IPV prevalence was highest among women aged 35–39 (27.6%) and women with primary education (32.4%). Regarding marital status, women who were cohabiting (37.6%), working women (28.0%), those without health insurance (26.0%), women with four or more children (28.2%), women with media exposure (27.0%), Christians (33.5%), women in the middle wealth index (30.6%), and those who belonged to the Igbo ethnic group (33.7%) had the highest proportion of experiencing IPV. The highest proportion of experiencing IPV was among women whose partners had primary education (31.7%), women whose partners were working (26.0%), women in polygamous marriages (27.3%), women living with partners (28.9%), women in rural areas (26.3%), women in the South South (36.2%), women who lived in communities with medium literacy levels (30.5%), women in medium community socioeconomic status (26.8%), and the 2018 survey year (33.2%).

### Random effects (variance measures) results

As indicated in Table 2, the AIC values reveal a reduction in Models I, II, and III, which included individual-level, interpersonal-level, and community-level variables, relative to Model IV, the final model. This notable drop in the models supports the fit quality of Model IV, which encompasses all the independent variables. Model IV is the comprehensive model chosen to predict factors related to IPV among women in Nigeria from 2008 to 2018. In the null model, significant differences were observed in the likelihood of factors linked to IPV among women in Nigeria across the clustering of the PSUs [σ² = 0.40, 95% CI 0.36–0.46]. The ICC value for Model 0 indicates that 10.90% of the variation in IPV among women of reproductive age in Nigeria was due to differences between clusters. This variation between clusters decreased to 9.78% in Model I, which included only individual-level variables. The ICC then rose to 10.62% in Model II, which contained interpersonal-level variables. The ICC decreased again to 10.11% in Model III, which focused solely on community-level variables. In the final model (Model IV), the variation between clusters further decreased to 8.95%. This can be linked to differences in the clustering of PSUs, which explains the variations in IPV among women of reproductive age in Nigeria from 2008 to 2018.

**Table 2:** Random effects (variance measures) results

Variable			Null model	Model I	Model II	Model III	Model IV
Random effect results							
PSU variance (95% CI)			0.40 (0.36–0.46)	0.36 (0.31–0.41)	0.39 (0.035–0.44)	0.37 (0.33–0.42)	0.32 (0.28–0.37)
ICC (100%)			10.90	9.78	10.62	10.11	8.95
Wald chi-square			Ref	1666.19***	400.09***	1859.82***	2813.89***
Model fitness							
Log likelihood			−26375.10	−25482.88	−26172.62	−25380.56	−24811.94
AIC			52754.19	51021.76	52357.25	50789.11	49711.87
BIC			52771.71	51266.99	52409.8	5091.73	50097.24
Weighted sample (N)			47,015	47,015	47,015	47,015	47,015
Number of groups			1,395	1,395	1,395	1,395	1,395

Source: 2008–2018 NDHSs

*AIC* Akaike’s Information Criterion, *aOR* adjusted odds ratio *BIC* Bayesian Information Criterion, *ICC* Intra-Class Correlation Coefficient, *PSU* Primary Sampling Unit, Ref-Reference category

^*^p < 0.05

^**^p < 0.01

^***^p < 0.001

### Multivariate analysis results on the factors associated with IPV among young women in Nigeria from 2008 to 2018

**Fig. 3:**
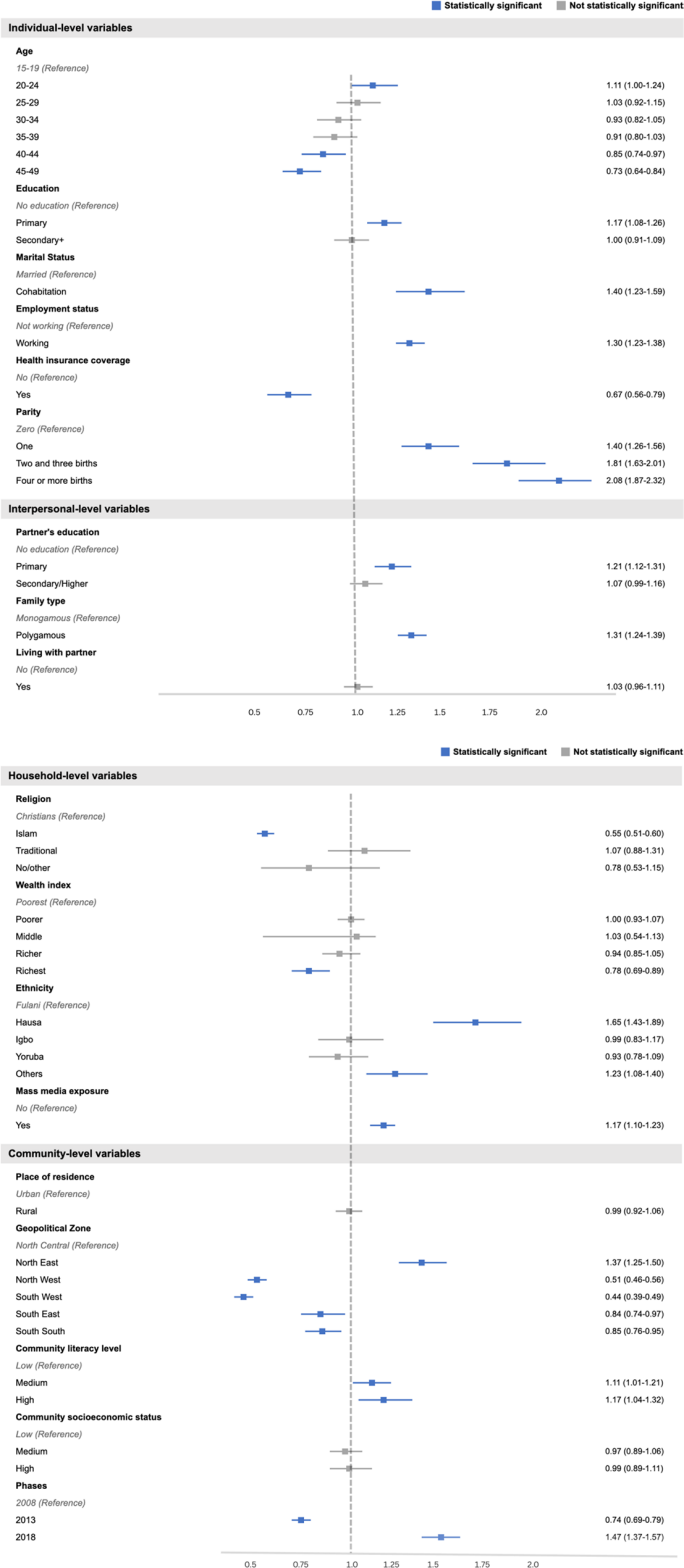
Forest plot of factors associated with intimate partner violence among Nigerian women (Model IV, adjusted odds ratios with 95% confidence intervals). Statistical significance: *p < 0.05, **p < 0.01, ***p < 0.001

Figure 3 shows the results of a multivariate analysis of the factors associated with IPV among women of reproductive age in Nigeria from 2008 to 2018. The final model (Model IV), which contains all the independent variables, revealed that women with primary education [aOR = 1.17; 95% CI = 1.08–1.26] and cohabiting women [aOR = 1.40; 95% CI = 1.23–1.59] had a higher likelihood of experiencing IPV compared to those who had no formal education and were married, respectively. Women who were working [aOR = 1.30; 95% CI = 1.23–1.59], with four or more births [aOR = 2.08; 95% CI = 1.87–2.32], women who were exposed to mass media [aOR = 1.17; 95% CI = 1.10–1.23], and those who belonged to the Hausa ethnic group [aOR = 1.65; 95% CI = 1.43–1.89] were more likely to experience IPV compared to those who were not working, had no births, were never exposed to mass media, and belonged to the Fulani ethnic group, respectively.

Furthermore, women whose partners had primary education [aOR = 1.21; 95% CI = 1.12–1.31], those in a polygamous family type [aOR = 1.31; 95% CI = 1.24–1.39], those who lived in the Northeast [aOR = 1.37; 95% CI = 1.25–1.50], those who lived in communities with high literacy levels [aOR = 1.17; 95% CI = 1.04–1.32], and those who were in the 2018 survey year [aOR = 1.47; 95% CI = 1.37–1.57] had higher odds of experiencing IPV than those in a monogamous family type, those in the North Central, those in communities with low literacy levels, and those in the 2008 survey year, respectively.

However, in terms of age, the likelihood of experiencing IPV was lower among women aged 45–49 [aOR = 0.73; 95% CI = 0.64–0.84] compared to those aged 15–19. Again, women who were covered by health insurance [aOR = 0.67; 95% CI = 0.56–0.79], Muslim women [aOR = 0.55; 95% CI = 0.51–0.60], and those in the richest wealth quintile IPV [aOR = 0.78; 95% CI = 0.69–0.89] were less likely to experience IPV compared to those who were not covered by health insurance, Christians, and those in the poorest wealth index, respectively.

## Discussion

We assessed the trend, prevalence, and determinants of IPV among women of reproductive age in Nigeria from 2008 to 2018. The results of this study show that IPV among women of reproductive age in Nigeria changed over the ten years from 2008 to 2018, with varying rates of occurrence and important social and demographic factors that affect the risk of IPV.

The trend analysis showed that sexual and emotional IPV had the highest recorded prevalence in 2018 at 4.51% [95% CI: 4.01%−5.07%] and 30.83% [95% CI: 29.48%−32.21%], respectively, whereas the highest prevalence of physical violence was recorded in 2008 at 13.96% [95% CI: 13.29%−14.66%]. This reveals the growing concern regarding sexual and emotional violence within intimate relationships. The average prevalence of emotional violence was 21.82% [95% CI: 21.03%−22.63%], whereas that of physical violence was 11.35% [95% CI: 10.86%−11.85%], and sexual violence was 3.53% [95% CI: 3.29%−3.80%]. The findings align with other studies conducted in Africa [26, 73], Iraq [74], and Nigeria [75], which indicate that emotional violence is the most prevalent form of intimate partner violence (IPV). Several factors contribute to its widespread occurrence, including controlling behaviours, relationship difficulties, and traditional gender roles [76]. Emotional intimate partner violence is frequently associated with negative mental and physical health outcomes, such as symptoms of depression and discomfort during sexual activity [77]. Hence, IPV in any form is a major public health issue affecting Nigerian women, and these findings are consistent with previous studies indicating that high IPV prevalence is shaped by multidimensional factors [1, 2, 7, 78].

We found that from 2008 to 2018, the overall prevalence of IPV among women of reproductive age in Nigeria was 25.94% [95% CI: 25.11%−26.80%]. Despite the various prevention strategies that have been instituted by the Nigerian government through the allocation of funds and other policies, such as the 2015 Violence Against Persons Prohibition (VAPP) Act, and access to support services, the IPV prevalence continues to be a serious public health issue [30]. The prevalence of IPV in this study is lower than 42.3% in Nigeria [79], 36% in Africa [9], 30.72% in Ethiopia [80], 39.23% in Gambia [81], 34% in Ghana [82], and 32.66% in East Africa [83].

The study’s multivariate regression analysis revealed that a variety of variables are risk factors associated with IPV among women of reproductive age in Nigeria. These risk factors are maternal education, marital status, employment status, parity, mass media exposure, ethnicity, partner’s educational level, family type, community literacy level, geopolitical zone, and survey year. The results of this investigation are in line with earlier studies from Africa [9, 26, 84], Nigeria [85], and Ghana [86]. However, maternal age, health insurance coverage, religion, and wealth quintile were identified as protective factors against IPV in Nigeria.

Studies conducted on IPV in Africa [84], India [87], and among 10 WHO multi-country [88] indicate that the relationship between education and the risk of IPV. It was determined through our analysis that women with primary education and those whose partners had primary education were more likely to experience IPV. This outcome is in line with several other studies from Africa [22, 84], Zambia [89], and Uganda [90]. One possible explanation for this observation is that partners who have higher education levels receive training that fosters a respect for human rights and gender equality, which subsequently motivates them to confront socio-cultural standards that diminish women’s status [84]. This highlights the importance of providing equal educational opportunities for both genders, which corresponds with SDG 4 that emphasizes quality education. However, women who lived in communities with high literacy levels were more likely to experience IPV.

Another key finding was the influence of marital status on women’s experience of IPV in Nigeria. Several studies have indicated a correlation between marital status and IPV [84, 91, 92]. The findings revealed that women who were cohabiting faced a greater likelihood of experiencing IPV compared to those who were married. Similar results have been obtained in Africa [66, 84] and China [93]. However, a study conducted in Uganda indicated that there was no significant difference in the risk of IPV between women who are cohabiting and those who are married [94]. The factors contributing to this increased risk are complex. One possible reason could be that those in marriage tend to have a better understanding of one another and are often more willing to make compromises on various matters, which leads to reduced conflict within their homes [84].

A significant and thought-provoking discovery from this research is that a woman’s employment status does not serve as a safeguard against IPV. Our finding is consistent with the findings of studies conducted in Africa [84, 95], multi-regionally [96], and in Nepal [97]. However, in a study of Mexican and American women, employment status was not a significant predictor of violence [98, 99]. The possible reason could be linked to a male reaction against shifting power structures in societies that have traditionally favoured men [100]. Nevertheless, the nature of employment is significant; women in higher-paying positions experience lower rates of IPV because women are more inclined to seek assistance and possess enhanced decision-making authority, which can help reduce IPV occurrences [101, 102]. These insights indicate that comprehensive empowerment approaches are essential for effectively mitigating IPV among working women.

In our analysis, it was discovered that women of reproductive age who had at least one birth are at higher risk of experiencing IPV. The study found that the odds of experiencing IPV increase with increasing parity. Specifically, women with four or more children were at greater risk of experiencing IPV. Similar results have been obtained in Nigeria [85] and Africa [103]. This may be due to the fact that women with multiple children often rely on their male partners for support, which could make them more vulnerable to IPV [85]. Additionally, women in those in a polygamous family type were more likely to experience IPV. This is confirmed by others [66, 104, 105]. The correlation varies by country and is affected by elements such as religion [105]. This study suggests that experiencing IPV is more common among women who are exposed to mass media. This finding is not in agreement with others conducted in India [106].

Research shows that there are notable ethnic disparities in the rates of IPV, with higher prevalence generally observed among ethnic minority groups [107, 108]. However, in our analysis, the odds of experiencing IPV were higher among the Hausa ethnic group. This finding is not in agreement with other studies conducted in Nigeria [32, 85, 109, 110] that revealed that women from the Yoruba ethnic group were more likely to experience IPV compared to those from the Hausa ethnic group. One possible explanation for this observation could be due to cultural norms that include patriarchal systems and a higher acceptance of gender inequality, lower levels of education and awareness regarding women’s rights, increased economic dependence that restricts options for leaving abusive situations, and possible underreporting by other ethnic groups that could distort the statistics.

Similar to other previous studies by Bolarinwa et al. [85] and Ezechi et al. [111], higher odds of IPV were experienced among women of reproductive age who resided in the Northeast. A possible reason could be due to the impact of the Boko Haram conflict, which exhibits a heightened susceptibility to IPV [112].

### Strengths and limitations

This study has several strengths. First, this study adds to the body of literature on IPV. Also, this study uses three waves of NDHS (2008, 2013, and 2018) to assess the trend and factors associated with IPV among women of reproductive age, providing a nationally representative and weighted analysis across a ten-year period. Again, the multilevel regression analysis applied accounted for the hierarchical structure of the DHS data, ensuring more reliable standard errors and estimates. Third, this study also allows for the generalization of the findings to other women in Nigeria and offers valuable insights for policymakers when designing and implementing effective IPV interventions.

Despite these strengths, this study has some weaknesses. First, the study relied on secondary data, and data for the analysis were limited to the variables found in the NDHS datasets. Hence, the interpretation should be limited to the variables that were used in this study. Additionally, the cross-sectional design of the survey does not allow for inference of causality from the findings. Since the DHS survey relied on self-reporting, there is a potential for recall bias, as well as the possibility that women provided socially desirable responses. Lastly, variables such as women’s knowledge and attitudes toward IPV could not be included in this analysis.

### Implications

Based on the findings of this study, there are considerable implications for policymakers, health practice, and future studies. The multifaceted relationship between individual, interpersonal, and community factors indicates that tackling IPV in Nigeria necessitates a holistic strategy. Policymakers are encouraged to develop layered approaches that not only support economic independence and higher levels of education for women but also confront ingrained socio-cultural norms that uphold gender disparities. Legal reforms that provide strong protections for women, particularly cohabiting women, along with community-based initiatives aimed at altering damaging attitudes and behaviours, can facilitate this process. From a health practice standpoint, broadening access to health insurance seems to offer protective benefits and can be a pathway to more extensive support networks. Community outreach initiatives to create awareness about IPV, especially in rural and low-literacy regions, should be prioritized to target vulnerable populations.

Further exploration of the intersectionality of variables like educational level, marital status, and economic conditions is vital for creating more adapted interventions. Longitudinal studies could provide in-depth perspectives into how shifts in socio-cultural and economic environments over time affect IPV rates. Additionally, qualitative research would be essential to understand the complex ways IPV is experienced and reported among various demographic groups, which could lead to more targeted policy responses.

Overall, the implications of this study advocate for collaborative efforts between government, healthcare providers, community leaders, and civil society to reduce IPV and assist affected women.

## Conclusion

The research indicates that intimate partner violence continues to be a significant public health concern in Nigeria, with approximately 25.94% of women of reproductive age experiencing IPV from 2008 to 2018, reaching a peak of 33.24% in 2018, which signals a troubling upward trend. The most common type of violence reported was emotional abuse, impacting nearly 22% of women. Major risk factors identified include lower educational attainment for both women and their partners, cohabitation, employment status, higher numbers of children (four or more), access to mass media, identification as Hausa, involvement in polygamous family arrangements, residing in the Northeast region, and living in areas with higher literacy rates. These findings highlight the multi-faceted factors contributing to IPV. The ongoing impact of sociodemographic and regional influences, along with the increasing rates of violence, indicates that current measures may not be sufficient. To effectively address this issue, it is necessary to implement targeted, multi-faceted strategies that focus on educational inequities, economic conditions, cultural practices, and regional disparities, with a strong focus on prevention to halt the rising trend of violence.

## Data Availability

The dataset is freely accessible via this link: [https://dhsprogram.com/data/dataset_admin/index.cfm].

## Abbreviations

AIC: Akaike’s Information Criterion (AIC)
AOR: Adjusted Odds Ratio
CI: Confidence interval
DHS: Demographic and Health Surveys
ICC: Intra-Class Correlation Coefficient
IPV: Intimate partner violence
NDHS: Nigeria Demographic and Health Surveys
PSU: Primary Sampling Unit
SEM: Socio-ecological Model
VIF: Variance Inflation Factor
WHO: World Health Organization
